# Developmental and Temperature-Driven Variations in Metabolic Profile and Antioxidant Capacity of Broccoli (*Brassica oleracea* var. *cymosa*)

**DOI:** 10.3390/plants14121825

**Published:** 2025-06-13

**Authors:** Daria Gmižić, Ivana Šola

**Affiliations:** Department of Biology, Faculty of Science, University of Zagreb, Horvatovac 102a, 10000 Zagreb, Croatia

**Keywords:** Brassicaceae, climate change, high temperature, mature broccoli, microgreens, metabolic response, phytochemicals, seedlings

## Abstract

This study investigates the impact of high temperature (HT) on the metabolic profile, oxidative-stress parameters, and antioxidant capacity of broccoli (*Brassica oleracea* var. *cymosa*) at different developmental stages—microgreens, seedlings, and two organs at the mature stage (leaves and head). We used spectrophotometric and chromatographic methods to quantify the concentrations of different groups and individual phenolic compounds, *L*-ascorbic acids, soluble sugars, proteins, glucosinolates, nitrates, pigments, oxidative-stress parameters, and antioxidant capacity. The highest number of analyzed variables significantly impacted by HT was in mature broccoli heads, with the most substantial change being an increase in proline by 168%. The lowest number of variables susceptible to HT (66%) was in the leaves of mature broccoli. The most dramatic change observed in this study was an increase in proline in seedlings by 587%. Statistical analyses showed that developmental stage plays a dominant role in shaping metabolic profiles, while HT further modulates it. Based on the measured parameters, the average contribution of developmental stage to the variance was 75%, while temperature explained 39% of the variance. The highest proportion of variance caused by temperature was seen in proline (92%), followed by kaempferol (80%), chlorophyll *a*/*b* (76%), soluble sugars (73%), total flavonoids (65%), antioxidant capacity measured by DPPH (58%), and chlorophyll/carotenoids ratio (56%). Temperature explained more variance than developmental stage for the concentration of soluble sugars, total hydroxycinnamic acids, and total tannins, which indicates an important role of these metabolites’ groups in the response of broccoli to HTs. The interaction of developmental stage and temperature explained more variance than developmental stage alone for the concentration of total proanthocyanidins, hydroxycinnamic acids, and phenolic acids. These findings underscore the complexity of metabolic regulation in broccoli and emphasize the importance of considering both developmental stage and environmental conditions when assessing its nutritional and functional properties.

## 1. Introduction

Temperature is one of the most important factors influencing plant growth, development, and metabolism [1]. Changes in environmental temperature lead to significant changes in plants on both molecular (proteins, nucleic acids) and supramolecular (membranes, cytoskeleton) levels [2]. Global temperatures are rising and, in the next few years, it is expected that the mean surface temperature will be up to 1.9 °C higher than the average over the years 1850–1900 [3]. Moreover, due to climate change, extreme heat waves are more frequent, longer, and more intense [4]. This presents a threat to food security as high temperatures (HTs) lead to reduced quality and lower yield in agricultural crops [5,6]. HTs can denature proteins and increase membrane fluidity and thereby disrupt processes like photosynthesis and nutrient transport [2]. Furthermore, HTs often lead to the increased production of reactive oxygen species (ROS), which can lead to oxidative stress and the damage of vital biomolecules [7]. However, ROS can act as signaling molecules and induce the synthesis of protective compounds [7,8]. Plants under HT stress accumulate osmolytes and specialized metabolites, including phenolic compounds and glucosinolates, as a mechanism to enhance stress tolerance [9,10]. The response of plants to HT differs between species and organs and depends on the intensity and duration of the HT [11].

Brassicaceae, also known as cruciferous plants, is one of the most economically important plant families, making up a big proportion of the human diet [12]. The most recognized species of Brassicaceae family are edible vegetables such as *Brassica oleracea* (broccoli, cabbage, cauliflower, kale), *B. rapa* (turnip), *B. nigra* (black mustard), *Raphanus sativus* (radish), and *Eruca sativa* (salad rocket) [13]. These species are nutritious and contain different phytochemicals with health benefits, including vitamins (C, E, folic acid), phenolic compounds, carotenoids, and glucosinolates [13,14]. The concentration of these bioactive compounds varies between cruciferous plants, as well as throughout the development of different organs, and is impacted by various environmental factors [15,16,17]. Recently, the consumption of younger developmental stages of cruciferous plants, such as sprouts, microgreens, and seedlings, has increased, and studies are showing that these earlier developmental stages often contain higher concentrations of bioactive phytochemicals than the adult plants [17,18,19]. For example, glucosinolates, nitrogen, and sulfur-containing specialized metabolites characteristic of the Brassicaceae family exhibit a decline in their concentration during plant development [20,21]. Their hydrolysis products, isothiocyanates, are important in the defense mechanisms of plants against various stressors, and numerous studies have confirmed their role in cancer prevention [13,14]. Furthermore, cruciferous plants are a great source of phenolic compounds, in which hydroxycinnamic acids and flavonoids are the most present in the *Brassica* genus [22,23]. These phytochemicals, mostly known for their antioxidant properties, also exhibit anti-inflammatory, antiproliferative, and antimicrobial activity and have been associated with numerous health benefits, including a reduced risk of chronic diseases such as cancer and cardiovascular disorders [22,23,24]. Their biosynthesis is often triggered under stressful conditions, such as HT, low temperature, drought, flood, and salinity, which highlights their role in stress resilience [25].

Since we rely on plants not only as a source of nutrition but also as a source of bioactive compounds that help to reduce the risk of diseases, it is important to investigate the effect of HTs on their phytochemical content and bioactivities. The concept of hormesis suggests that heat stress can induce adaptive responses in plants, leading to an increased concentration of phytochemicals that have potential health benefits [26]. Studies have shown that HTs increased the content of glucosinolates [20] and phenolic compounds [27] in broccoli sprouts. A study on mature cabbage and kale showed that HTs increased their antioxidant potential; however, it increased the phenolic content in cabbage only [28]. These findings highlight the complexity of plant response to HT and underscore the need to analyze different organs and developmental stages to better understand adaptive mechanisms and potential consequences for human health.

This study aimed to investigate the effects of HT on metabolic profiles, oxidative-stress parameters, and antioxidant capacity throughout the development of broccoli (*Brassica oleracea* L. convar. *botrytis* (L.) Alef. var. *cymosa* Duch.). The analysis focused on three developmental stages, those of microgreens, seedlings, and mature plants, as well as different plant organs (leaves and heads) at the mature stage. We measured the content of different groups as well as individual phytochemicals, oxidative stress parameters, and the antioxidant capacity of broccoli in order to assess the changes that happen throughout the development of broccoli and compare the responses of different developmental stages to HT stress. Our hypothesis was that different developmental stages and organs would show specific phytochemical responses to HT. The results of this study contribute to a better understanding of plant adjustment to HTs, reveal the direction of phytochemical changes during plant development, and emphasize which type of phytochemicals in each of developmental stage is predominantly affected by HT.

## 2. Results and Discussion

Plants produce a wide range of metabolites throughout their development, which are essential for their growth, reproduction, and interaction with the environment [29]. The concentration of these metabolites varies depending on the developmental stage, organ type, and environmental conditions [16]. Metabolic plasticity enables plants to dynamically reprogram their metabolic pathways in response to stress, leading to the accumulation of stress-related metabolites such as osmolytes, antioxidants, and signaling molecules [30]. Understanding how the metabolic profile of plants changes during development and in response to HT is key for improving crop resilience and nutritional quality, especially in the context of climate change. In this study, we investigated the metabolic profile and antioxidant capacity of broccoli samples across different developmental stages and two organs at the mature stage and evaluated the impact of HT on them.

### 2.1. Polyphenolics in Broccoli Throughout Development and in Response to HT

Phenolic compounds are specialized metabolites that play a crucial role in plant development and stress adaptation [23,25]. They are involved in diverse physiological processes such as cell wall formation, pigmentation, and the regulation of plant growth [31]. Their concentration changes throughout development [17] as well as in response to stressful conditions such as extreme temperatures, drought, and floods [32,33,34]. These compounds contribute not only to plant development and stress response but also to their nutritional and health-promoting properties for consumers, mainly due to their high antioxidant activity [8,23].

#### 2.1.1. Groups of Phenolic Compounds

**Figure 1:**
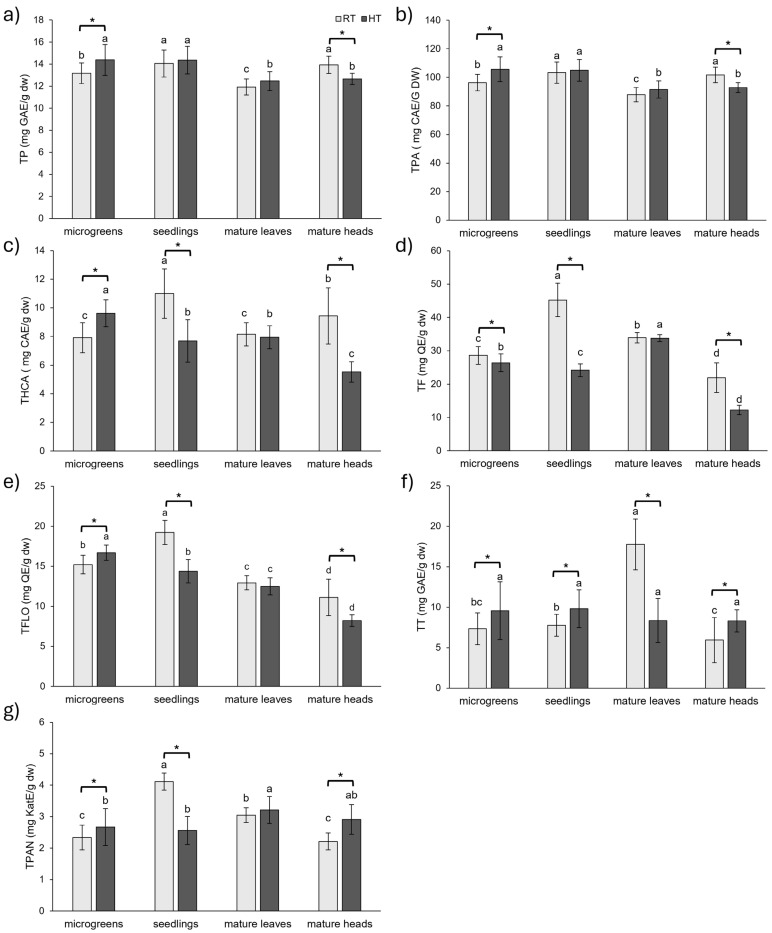
The concentration of total (**a**) phenolics (TP), (**b**) phenolic acids (TPA), (**c**) hydroxycinnammic acids (THCA), (**d**) flavonoids (TF), (**e**) flavonols (TFLO), (**f**) tannins (TT), and (**g**) proanthocyanidins (TPAN) during development and in different organs at the mature stage of broccoli (*Brassica oleracea* var. *cymosa*) grown at room (RT) or high (HT) temperature. Values represent the mean ± standard deviation of three (mature plants) or six (microgreens, seedlings) biological replicates and four technical replicates. An asterisk (*) indicates a significant difference between plants grown at HT and RT within the same developmental stage or organ at the mature stage (Student’s *t*-test, *p* ≤ 0.05). Different letters indicate significant differences among developmental stages or organs grown under the same temperature (one-way ANOVA, Duncan’s test, *p* ≤ 0.05). CAE = caffeic acid equivalents; GAE = gallic acid equivalents; KatE = catechin equivalents; QE = quercetin equivalents.

High temperatures significantly impacted the concentration of all analyzed phenolic groups in broccoli microgreens and mature heads, suggesting that these tissues are the most responsive to HT at the polyphenolic level (Figure 1). In microgreens, HT increased the concentration of all analyzed phenolic groups, with the exception of flavonoids, which declined (−8%). The most substantial increases in microgreens were observed for total tannins (30%) and total hydroxycinnamic acids (22%) compared to the microgreens grown at RT. In contrast, an almost completely opposite response could be observed in mature broccoli heads. HT decreased the concentration of most phenolic groups, except total tannins and total proanthocyanidins, which increased. Broccoli heads displayed a stronger response to HT than microgreens, with five out of seven analyzed phenolic groups changing by more than 20% of the value obtained from heads grown at RT. Notably, flavonoids decreased by 44% and hydroxycinnamic acids by 41%, while total tannins increased by 40% and proanthocyanidins by 31%. In contrast, total tannins were the only group of phenolics significantly affected by HT in the leaves of mature broccoli, with their concentration dramatically declining by 53%. This suggests that the leaves of mature broccoli display a less diverse response to HT in terms of groups of phenolic compounds compared to the other developmental stages. In seedlings, the change in total phenolic compounds and total phenolic acid content under HT conditions was not statistically significant. However, the other five groups of phenolics responded strongly to HT, with changes exceeding 25% compared to RT. Flavonoids decreased by 47%, proanthocyanidins by 38%, hydroxycinnamic acids by 30%, and flavonols by 25%. Contrarily, tannins increased by 26%.

Taken altogether, HT promoted the accumulation of different phenolic groups at the microgreens stage, with the exception of flavonoids, which declined. In contrast, the seedlings and heads of mature broccoli were predominantly negatively affected by HT, exhibiting greater decreases in most phenolic groups and a stronger overall response to HT compared to microgreens and mature leaves. Total tannins significantly changed under HT conditions in all stages and organs (increased in microgreens, seedlings, and mature heads and decreased in mature leaves), highlighting their role in the stress response of broccoli. At the level of polyphenolic groups, leaves of mature broccoli were the most HT-resistant organ of broccoli, with significant change detected for total tannins only. Most previous studies focused on the effects of HT on total phenolic content in different species or genotypes at a single developmental stage only. For instance, in radish sprouts, HT increased total phenolics in the cultivated variety but had no significant effect in the wild type [14]. Similarly, an increase in total phenolics was reported in cabbage grown at 32 °C compared to 20 °C, while there was no statistical difference found between kale plants under the same conditions [28]. Similarly, one-month exposure to 35 °C increased total phenolics in two-month-old tomato (*Lycopersicon esculentum*) plants but decreased them in watermelon (*Citrullus lanatus*) plants [35]. In addition, short-term heat shock of 45 °C and 50 °C for 1 h daily over a week had a negative impact on total phenolics and flavonoids in one-month-old tomato (*Solanum lycopersicum*) plants [36]. Our study expands on these findings by demonstrating that divergent phenolic responses to HT are not limited to interspecies variability but can also manifest within a single species, depending on the developmental stage and organ. These insights underscore the importance of stage- and organ-specific analyses when evaluating the effects of abiotic stress on the phytochemical profile of crop plants.

Under RT conditions, total phenolics and phenolic acids showed the highest concentrations in broccoli seedlings and mature heads, with lower levels in microgreens and the lowest concentrations in mature leaves. For flavonoids, seedlings exhibited the highest concentration, followed by mature leaves, microgreens, and mature heads. Hydroxycinnamic acids were the most abundant in seedlings, followed by mature heads, with microgreens and mature leaves showing the lowest levels. Flavonols displayed the highest concentration in seedlings, followed by microgreens, mature leaves, and mature heads. Tannins were the most concentrated in mature leaves, while proanthocyanidins showed their highest level in seedlings, followed by mature leaves, microgreens, and mature heads. For comparison, in a study of three different cultivars of *B. oleracea*, the concentration of total phenolics decreased with the age of the plants, with the highest amount found in sprouts and the lowest in plantlets with almost three true leaves [22]. Conversely, another study on broccoli (*B. oleracea* var. *italica* cv. ‘Legacy’) sprouts of varying ages reported a continuous increase in total phenolics from day 3 to day 11, although flavonoid concentrations showed no continuous trend [37]. The highest concentration of flavonoids was found in 11-day-old sprouts, while the lowest was found in 3-day-old sprouts. However, flavonoid concentration was higher in 8-day-old sprouts than in 5-day-old sprouts [37]. For comparison, in a study of choy sum (*B. rapa* var. *parachinensis*), a higher concentration of phenolic compounds was observed in 15-day-old plants (with three true leaves) than in 7-day-old plants (with the first true leaf) and 30-day-old plants (adult stage) [23].

Under HT conditions, total phenolics and phenolic acids reached their highest level in the early developmental stages (microgreens and seedlings), while mature plants (leaves and heads) exhibited lower concentrations. Flavonoids were the highest in mature leaves, with lower levels in microgreens, followed by seedlings, and the lowest in mature heads. Hydroxycinnamic acids and flavonols showed a similar trend during the development of broccoli under HT conditions, with their concentration declining with the age of the plant. The concentration of hydroxycinnamic acids and flavonols was the highest at the microgreens stage and the lowest at the mature stage, with heads having the lowest concentration of these groups of phenolics. The total tannin concentration was stable during the development and in different organs at the mature stage, with no statistical difference found between analyzed stages/organs. Proanthocyanidins were also stable throughout the development of broccoli exposed to HT, apart from mature broccoli leaves, which had significantly higher amounts than in earlier developmental stages.

#### 2.1.2. Individual Phenolic Compounds

The most prominent polyphenols in *Brassica* species are flavonoids and hydroxycinnamic acids [22,23]. Flavonoids and hydroxycinnamic acids commonly found in *Brassica* species, like kaempferol, quercetin, sinapic, and ferulic acid, are mostly found in conjugated forms [8,16]. Therefore, we performed acidic hydrolysis to analyze the concentration of these aglycones in broccoli throughout its development and in two organs at the mature stage, as well as the impact of HT on their concentration.

**Figure 2:**
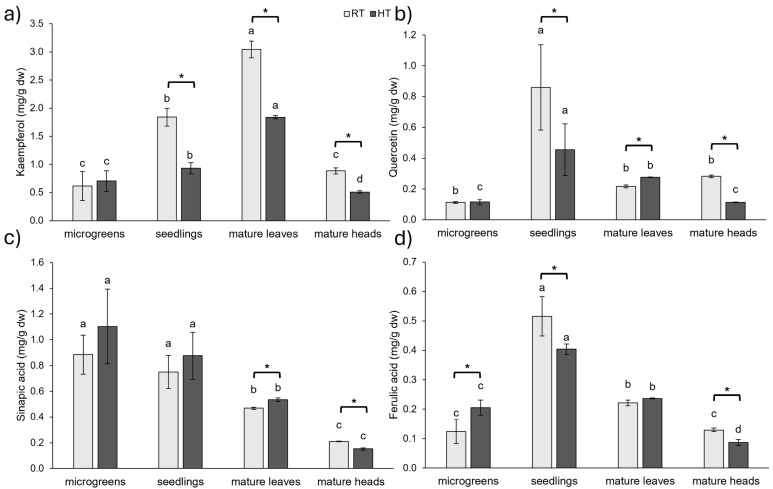
The concentration of (**a**) kaempferol, (**b**) quercetin, (**c**) sinapic acid, and (**d**) ferulic acid during development and in different organs at the mature stage of broccoli (*Brassica oleracea* var. *cymosa*) grown at room (RT) or high (HT) temperature. Values represent the mean ± standard deviation of three (mature plants) or six (microgreens, seedlings) biological replicates and four technical replicates. An asterisk (*) indicates a significant difference between plants grown at HT and RT within the same developmental stage or organ at the mature stage (Student’s *t*-test, *p* ≤ 0.05), while different letters indicate significant differences among developmental stages or organs grown under the same temperature conditions (one-way ANOVA, Duncan’s test, *p* ≤ 0.05).

Kaempferol concentration was negatively influenced by HT from the stage of seedlings onward (Figure 2a). The most substantial decline was observed in seedlings (−49%), followed by mature broccoli heads (−43%) and mature leaves (−40%). In contrast, at the microgreens stage, kaempferol concentration increased by 15%, but this increase was not statistically significant. Similar to our results for microgreens, in another study, broccoli (*B. oleracea* var. *italica* cv. ‘Ramoso Calabrese Tardivo’) sprouts grown at 30 °C had a higher concentration of kaempferol derivatives than sprouts grown at 20 °C, but this increase, at the level of *p* ≤ 0.05, was not statistically significant [27]. However, in the same study, rocket (*Eruca sativa* cv. ‘A Foglia Lobata’) sprouts grown at 30 °C showed a significant increase in kaempferol derivatives compared to sprouts grown at 20 °C [27].

Quercetin concentration was also negatively impacted by HT, with reductions by 47% in seedlings and 60% in mature broccoli heads (Figure 2b). However, in mature broccoli leaves, HT led to a 27% increase in quercetin content. At the microgreens stage, a slight increase in quercetin content was observed, although this was not statistically significant. Other studies have also investigated the impact of temperature on kaempferol and quercetin content. For example, one study examined the impact of temperature and photoperiod on broccoli (*B. oleracea* var. *italica* cv. ‘Lord’) florets and found that higher temperature (18 °C, compared to 12 °C as the low-temperature condition) led to increased concentrations of both flavonoids, regardless of photoperiod length [38].

Sinapic acid concentration showed a trend of increasing in microgreens and seedlings and a significant increase in mature leaves under HT conditions (14%), while, in mature heads, the concentration decreased by 28% (Figure 2c). Although the highest increase in sinapic acid under HT conditions was found at the microgreens stage (25%), followed by an increase of 17% at the seedlings stage, this was not statistically significant due to high variability in the samples.

Ferulic acid increased with HT by 66% at the microgreens stage, while it decreased by 22% at the seedlings stage and 33% in the mature broccoli heads (Figure 2d). The slight increase in ferulic acid concentration in broccoli leaves grown at HT was not significant. In our previous research, HT increased the concentration of both conjugated and free ferulic acid in young broccoli plants that were 15–20 days old, which corresponds to the microgreens stage in this study [34,39,40]. Furthermore, heat shocks of 45 °C and 50 °C for 1 h over 7 days led to increased concentrations of ferulic acid in one-month-old tomato seedlings [36].

As shown in Figure S1, HT significantly affected the concentration of total identified phenolics (TIP) across all developmental stages and organs at the mature stage. TIP concentration decreased under HT conditions by 43% in the heads of mature broccoli, 33% in seedlings, and 27% in the leaves of mature broccoli. On the other hand, at the microgreens stage, the concentration of TIP was increased under HT conditions by 23%.

The concentration of individual phenolic compounds varied throughout the development of broccoli under both growth temperatures (Figure 2). Kaempferol levels tended to increase with development, with the highest levels found in mature leaves, followed by seedlings. Lower levels of kaempferol were found at the microgreens stage and in broccoli heads. The fact that kaempferol concentration peaked in mature leaves under both RT and HT conditions suggests its potential role in photosynthesis processes [41]. Quercetin concentration remained relatively stable throughout the development of broccoli grown at RT, although a pronounced peak was observed in seedlings under both RT and HT conditions. Similarly, ferulic acid concentration peaked in seedlings under both temperature conditions, indicating an important role of these polyphenolics at the seedling stage of broccoli. On the other hand, sinapic acid concentration declined as broccoli matured, with the highest concentration detected at the microgreens stage and the lowest concentration in mature heads. This is in concordance with a study on choy sum in which a decline in sinapic acid derivatives was observed from sprout to adult stage [23]. Furthermore, similar findings were reported in a study examining developmental changes in Chinese cabbage (*B. rapa* ssp. *pekinensis*). In that study, the concentration of sinapic acid declined and the concentration of kaempferol increased with plant age, while quercetin levels remained relatively stable throughout development [17]. Likewise, another study on two cultivars of broccoli (*B. oleracea* var. *italica* cv. ‘Cavolo Broccolo Ramoso Calabrese’ and *B. oleracea* var. *italica* cv. ‘Broccolo Nero’) and one kale cultivar (*B. oleracea* var. *acephala* cv. ‘Cavolo Lacinato Nero di Toscana’) revealed that kaempferol levels increased with age in both cultivars of broccoli [22], which is consistent with our results. However, in kale, kaempferol levels decreased from the sprout to the microgreen stage and then increased at the stage when plants had almost three true leaves, but with levels being lower than at the sprout stage [22].

Under RT conditions, the highest concentration of total identified phenolics (TIP) was observed at the seedlings stage and in mature leaves (3.96 ± 0.37 and 3.95 ± 0.17 mg/g dw, respectively), while lower values were recorded in microgreens and mature heads (1.74 ± 0.16 and 1.51 ± 0.05 mg/g dw, respectively) (Figure S1). Under HT conditions, TIP concentrations differed significantly across all developmental stages and organs. The highest TIP concentration was recorded in the mature leaves (2.88 ± 0.03 mg/g dw), followed by seedlings (2.67 ± 0.17 mg/g dw), microgreens (2.13 ± 0.09 mg/g dw), and mature heads (0.86 ± 0.03 mg/g dw) (Figure S1). These results further support the notion that phenolic composition and concentration in broccoli are strongly influenced by both developmental stage and organs at the mature stage and that HTs act as an additional modulating factor.

### 2.2. L-Ascorbic Acid

Broccoli is an excellent source of vitamins, including vitamin C (also known as ascorbic acid), which acts as an antioxidant and helps plants under stressful conditions [42]. In this study, we analyzed the concentration of *L*-ascorbic acid during development and across two organs at the mature stage of broccoli and the impact of HTs on it.

**Figure 3:**
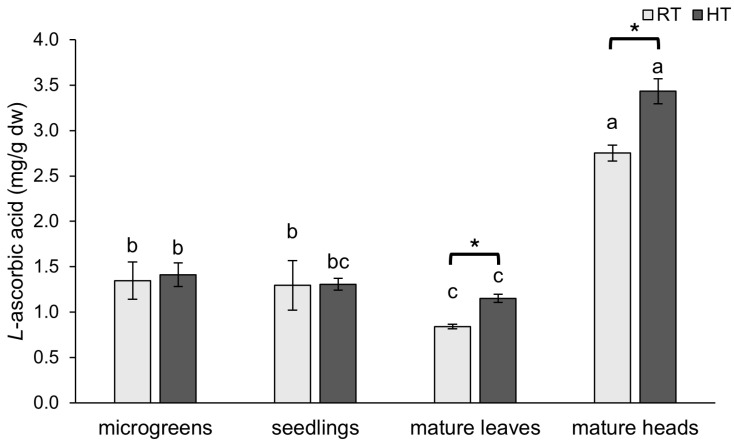
The concentration of *L*-ascorbic acid during development and in different organs at the mature stage of broccoli (*Brassica oleracea* var. *cymosa*) grown at room (RT) or high (HT) temperature. Values represent the mean ± standard deviation of three (mature plants) or six (microgreens, seedlings) biological replicates and four technical replicates. An asterisk (*) indicates a significant difference between plants grown at HT and RT within the same developmental stage or organ at the mature stage (Student’s *t*-test, *p* ≤ 0.05), while different letters indicate significant differences among developmental stages or organs grown under the same temperature conditions (one-way ANOVA, Duncan’s test, *p* ≤ 0.05).

The concentration of *L*-ascorbic acid was significantly increased under HT conditions in mature broccoli plants (Figure 3). In mature leaves, the concentration of *L*-ascorbic acid was boosted by 37%, while, in mature heads, it was boosted by 25% compared to broccoli grown under RT conditions. Similarly, in a study on a tomato plant exposed to HT, of 32 °C at different reproductive stages, it was found that HT had a positive impact on the concentration of *L*-ascorbic acid during flowering and early fruit developmental stages, while, at advanced stages of fruit development, the concentration was negatively impacted [43]. In earlier developmental stages of broccoli (microgreens and seedlings), the concentration of *L*-ascorbic acid was not affected by HT. Likewise, in both broccoli (*B. oleracea* var. *italica*, cv. ‘Ramoso Calabrese Tardivo’) and rocket (*Eruca sativa* cv. ‘A Foglia Lobata’) sprouts, *L*-ascorbic acid concentration was not changed when grown at 30 °C compared to 20 °C [27]. Moreover, the resistance of ascorbic acid content was observed in tomato seedlings exposed to heat stress of 40 °C for 8 days as well [44].

The concentration of *L*-ascorbic acid followed a similar pattern throughout the development of broccoli plants under both HT and RT conditions. The highest concentration was in the mature broccoli heads and the lowest was in the mature broccoli leaves. This is in concordance with a study of the nutritional composition of different broccoli (*B. oleracea* var. *italica*) parts, in which it was found that broccoli flower buds have a higher concentration of vitamin C than leaves [45]. The microgreens and seedlings stages had higher levels of *L*-ascorbic acid than the leaves of mature broccoli. However, there was no significant difference between the microgreen and seedling stages as the *L*-ascorbic acid concentration remained relatively stable. Similar results were reported for a different broccoli variety (*B. oleracea* var. *italica* cv. ‘Cavolo Broccolo Ramoso Calabrese’) in which the concentration of ascorbic acid was relatively stable from sprouts to young plants with nearly three true leaves [22]. In contrast, in a different broccoli variety (*B. oleracea* var. *italica* cv. ‘Broccolo Nero’), the concentration of ascorbic acid was higher at the microgreens stage than at the sprout and baby leaf stages. On the other hand, the concentration of *L*-ascorbic acid was the highest at the youngest developmental stage of Chinese cabbage, after which, it remained relatively stable throughout development [17]. In contrast, kale exhibited an increase in ascorbic acid throughout its early development [22].

### 2.3. Soluble Sugars

Sugars play a crucial role in plant response to abiotic stress, such as extreme temperatures, drought, and salinity. They function both as an energy source and as a skeleton for building specialized plant metabolites [46]. They are considered osmolytes and help plants to stabilize water content and protect cells from dehydration [47]. Furthermore, they also contribute to membrane and protein stabilization and can act as signaling molecules by inducing the expression of genes related to stress response [46].

**Figure 4:**
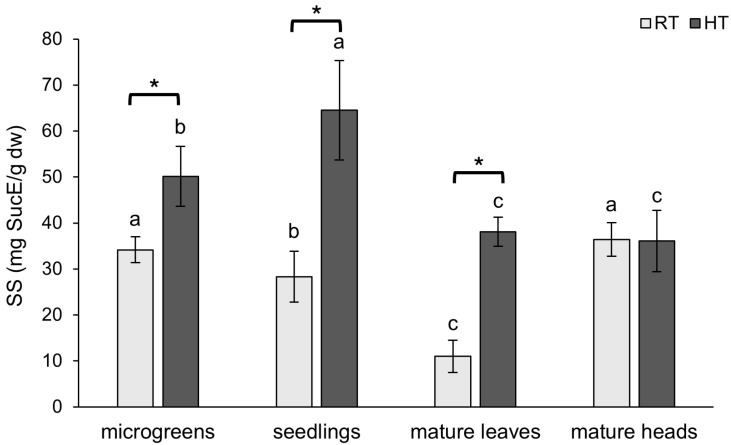
The concentration of soluble sugars (SS) during development and in different organs at the mature stage of broccoli (*Brassica oleracea* var. *cymosa*) grown at room (RT) or high (HT) temperature. Values represent the mean ± standard deviation of three (mature plants) or six (microgreens, seedlings) biological replicates and four technical replicates. An asterisk (*) indicates a significant difference between plants grown at HT and RT within the same developmental stage or organ at the mature stage (Student’s *t*-test, *p* ≤ 0.05), while different letters indicate significant differences among developmental stages or organs grown under the same temperature conditions (one-way ANOVA, Duncan’s test, *p* ≤ 0.05). SucE = sucrose equivalents.

An important role of soluble sugars in plant response to HT can be observed in this study as well. Their concentration was markedly increased in all analyzed tissues grown in HTs, except in mature broccoli heads (Figure 4). The highest increase with HT in soluble sugar content was found in the leaves of mature broccoli, in which the concentration increased by 246% of the value of broccoli grown at RT. Broccoli microgreens and seedlings grown at HT had SS boosted by 47% and 128%, respectively. The accumulation of soluble sugars under HT conditions was also observed in our previous works [39,40,48]. A similar gradual increase in soluble sugars with days grown in HTs was reported in a heat-tolerant ‘268’ line of one-month-old Chinese cabbage plants exposed to HT of 40 °C for 8 days [49]. Moreover, the accumulation of soluble sugars was more evident in two heat-tolerant genotypes of wheat than in heat-sensitive genotypes of wheat [50], suggesting their crucial role in mitigating the adverse effects of HT. Furthermore, gradual increases in glucose, fructose, and sucrose with days exposed to HTs of 30 °C were also reported for the leaves of two-month-old oilseed rape (*B. napus*) plants [51]. The accumulation of soluble sugars in response to HT was reported in four different varieties of leaf lettuce (*Lactuca sativa*) seedlings [52], as well as in blueberry (*Vaccinium corymbosum*) leaves [53]. We suppose that the accumulation of SS makes plants more heat tolerant by improving osmotic regulation and ROS scavenging and providing the building structure for the synthesis of specialized metabolites.

The highest concentration of soluble sugars in broccoli plants grown at RT was found in mature broccoli heads and microgreens. The role of sugars in initiating flowering in some species [54] may explain the higher concentration of soluble sugars in broccoli heads compared to leaves of the same plants. Slightly lower concentrations were observed in seedlings grown at RT, while the lowest concentration was found in the leaves of mature broccoli at RT. We assume that the sugars are removed from the place of their synthesis to provide enough energy and supplies for all parts of the plant and enable further synthesis in leaves. In broccoli grown in HTs, the highest concentration of soluble sugars was observed in seedlings, while the leaves and heads of mature broccoli exhibited the lowest concentrations, which did not significantly differ from each other.

### 2.4. Nitrogen-Containing Phytochemicals

**Figure 5:**
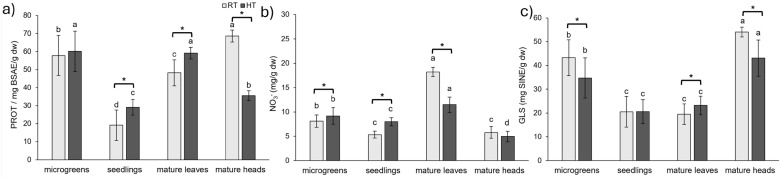
The concentration of (**a**) soluble proteins (PROT), (**b**) total nitrates (NO_3_^−^), and (**c**) total intact glucosinolates (GLS) during development and in different organs at the mature stage of broccoli (*Brassica oleracea* var. *cymosa*) grown at room (RT) or high (HT) temperature. Values represent the mean ± standard deviation of three (mature plants) or six (microgreens, seedlings) biological replicates and four technical replicates. An asterisk (*) indicates a significant difference between plants grown at HT and RT within the same developmental stage or organ at the mature stage (Student’s *t*-test, *p* ≤ 0.05), while different letters indicate significant differences among developmental stages or organs grown under the same temperature conditions (one-way ANOVA, Duncan’s test, *p* ≤ 0.05). BSAE = bovine serum albumin equivalents; SINE = sinigrin equivalents.

Soluble proteins not only serve as a storage form of nitrogen but are also regarded as osmotic regulators in plants, helping to protect them from abiotic stresses, especially heat and drought [47]. HT increased the concentration of soluble proteins in broccoli seedlings by 52% and in the leaves of mature broccoli by 23%; however it decreased the concentration of soluble proteins in broccoli heads by 48% (Figure 5a). The increase of 4% by HT in the concentration of soluble proteins in broccoli microgreens was not statistically significant. An increase in soluble proteins was observed in *in vitro* cultures of *Thymus lotocephalus* exposed to 30 °C for 7 weeks [47], as well as in a heat-tolerant cultivar (‘Amalia’) and wild thermotolerant type (‘Nagcarlang’) of tomato plants exposed to 45 °C for 3 h [55]. In contrast, no significant change was observed in a thermosusceptible cultivar (‘Campbell-28’) of tomato grown under the same conditions [55], which suggests that the accumulation of soluble proteins plays an important role in heat stress tolerance in certain tomato genotypes, such as ‘Amalia’ and ‘Nagcarlang’. On the other hand, the concentration of proteins decreased in *in vitro* cultures of *Lavandula viridis* exposed to 30 °C for 7 weeks [47], as well as in one-month-old tobacco (*Nicotiana tabacum*) plants exposed to HTs (28.5 °C for 15–45 days) [56].

The highest concentration of soluble proteins in the RT group was observed in mature broccoli heads, whereas, in the HT group, mature leaves and microgreens exhibited the highest protein concentrations. In contrast, the lowest protein concentrations were consistently found in broccoli seedlings in both the RT and HT groups. In a study on tobacco plants, the concentration of proteins decreased from 30- to 90-day-old tomato plants across various temperature treatments [56].

Nitrate accumulation in plants is influenced by various abiotic factors, including temperature, light intensity, water availability, and nitrogen fertilization [57]. High nitrate intake from plants can lead to health risks, emphasizing the need to optimize agricultural practices to manage nitrate levels in crops [58]. Nitrates increased by HT in younger developmental stages (microgreens and seedlings), with their concentration increasing by 13% and 49%, respectively (Figure 5b). The opposite effect of HT on nitrates content was observed in mature broccoli plants. Their concentration diminished in the leaves by 37%, while the decrease of 15% in the heads was not statistically significant. In a study on two spinach cultivars (*Spinacia oleracea* cvs. ‘Northland’ and ‘Virginia Savoy’), one-month-old plants were exposed to different temperatures for an additional month, during which, an increase in temperature resulted in a higher nitrate accumulation [59] These findings, together with our results, suggest that the duration of exposure to HTs may play an important role in nitrate accumulation. In our experiment, seedlings (exposed to HTs for 22 days) showed the greatest increase in nitrate content, followed by microgreens (8 days), while mature plants (leaves and heads) exposed to HTs for only 5 days showed a reduction. This implies that prolonged exposure to HTs may enhance nitrate accumulation in earlier developmental stages, while mature organs may be less responsive or subject to nitrate reduction mechanisms.

Both the RT and HT groups showed a similar trend in the effect on the concentration of nitrates throughout the development of broccoli and different organs at the mature stage, with the highest concentration in the leaves of mature broccoli, followed by microgreens. Seedlings and mature broccoli heads had significantly lower concentrations of nitrates compared to those of the leaves of mature broccoli and broccoli microgreens. A study on various vegetables found that leafy crops accumulate significant amounts of nitrate even without nitrogen fertilization [60]. Older, outer leaves of cabbage contain more than twice the nitrate content of younger, inner leaves. Since nitrates are mainly transported through the xylem, which is dependent on transpiration, their content is low in plant parts that rely on material transported through phloem and do not transpire [60]. Therefore, the high concentrations of nitrates in the mature leaves can be explained by their longer exposure to nitrates and their storage in the vacuoles. In contrast, the lower nitrate content in the heads can be attributed to their reliance on phloem-transported material.

Glucosinolates are nitrogen- and sulfur-containing specialized plant metabolites found almost exclusively in the Brassicaceae family that give them a pungent flavor [14]. Their concentration is variable and depends on developmental stage, tissue, and exogenous factors [13]. Their degradation products are important defense mechanisms for plants against various stressors and are reported to have anticancerogenic activities in various models [14].

In our study, HT decreased the concentration of total intact glucosinolates in broccoli microgreens (−20%) and mature heads (−20%) and increased it in mature leaves (19%) (Figure 5c). The concentration of total glucosinolates in broccoli seedlings was not affected by HT. A decrease in glucosinolates in response to HT was observed in our earlier studies on broccoli seedlings and microgreens [39,40], as well as in the study on *B. oleracea* core collection during early development, i.e., seedling stage [61]. On the other hand, an increase in aliphatic and indole glucosinolates in *B. rapa* seedlings in response to short-term HT stress of 40 °C for 8 h [62] and an increase in total glucosinolates in broccoli (*B. oleracea* var. *italica* cv. ‘Marathon’) sprouts grown at 30 °C for 6–11 days were observed [20]. Both broccoli plants grown at RT and HT showed a similar trend in the concentration of total glucosinolates with plant development. The highest concentration was observed in mature heads, followed by microgreens, while a lower concentration was observed in seedlings and mature leaves. This is consistent with a study of three different *B. oleracea* varieties in which the highest concentration of total glucosinolates was found in the leaves of the youngest plants sampled 14 days after sowing [15]. The leaves of plants sampled 28 days after sowing had much lower concentrations of total glucosinolates and, after that, the concentration varied but remained lower than in the youngest plants. Furthermore, the concentration of glucosinolates in *Brassica* heads was the highest at the start of head formation, and the concentration was higher than in leaves, which is consistent with our results [15]. In 6–11-day-old broccoli sprouts, the highest concentration of glucosinolates was found in the youngest sprouts, and the concentration decreased with sprout maturity [20]. Moreover, the decline in glucosinolate concentration with sprout maturity was observed under both HT and optimum temperature conditions [20]. Also, in a study of nine different broccoli cultivars, it was shown that the concentration of total glucosinolates decreased from seed to 17-day-old plants [21].

### 2.5. Photosynthetic Pigments

Photosynthesis occurs across the thylakoid membranes and stroma of chloroplasts and is a crucial process through which plants convert light into chemical energy, which is stored in compounds. Since membranes are sensitive to temperature changes due to their structure, photosynthesis itself is affected by temperature changes. HTs increase the fluidity of the membrane itself and can inactivate many chloroplast enzymes [2,63]. They also cause many structural and functional disorders of chloroplasts, mainly due to the oxidative stress caused by HTs, which, as a result, leads to a reduction in photosynthesis rates [63]. The amount of chlorophyll indicates the photosynthetic capacity of plants and is influenced by the various environmental stressors, including extreme temperatures [64].

**Figure 6:**
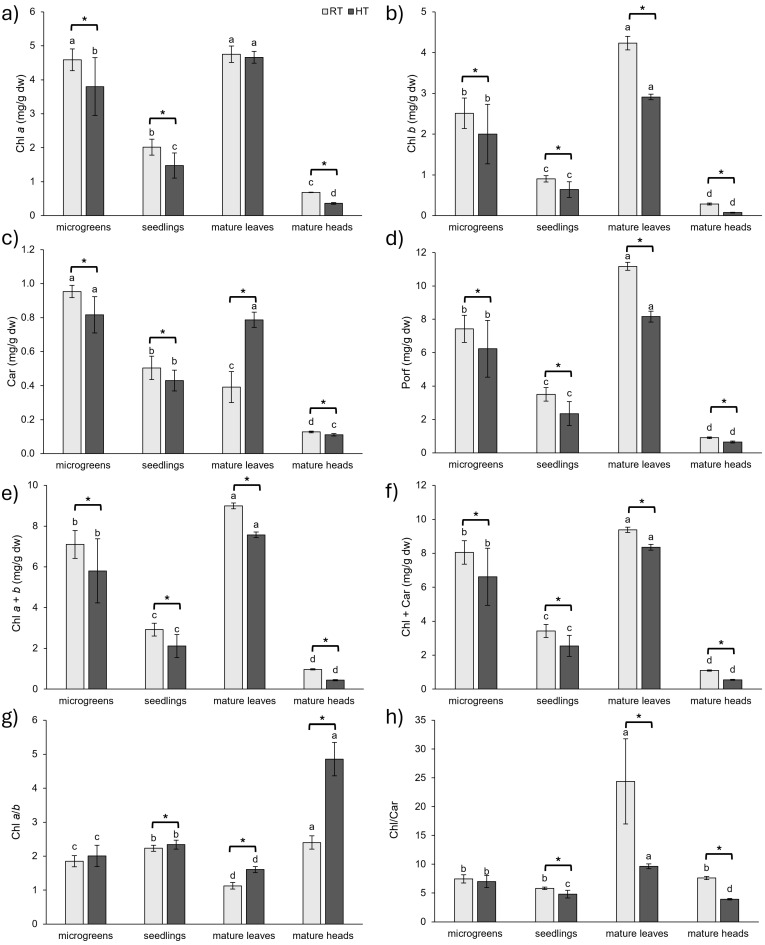
The concentration of (**a**) chlorophyll *a*, (**b**) chlorophyll *b*, (**c**) carotenoids (Car), (**d**) porphyrins (Porf), (**e**) total chlorophyll (Chl), (**f**) total chlorophyll and carotenoid, the ratio of (**g**) chlorophyll *a*/*b*, and (**h**) total chlorophyll/total carotenoids during development and in different organs at the mature stage of broccoli (*Brassica oleracea* var. *cymosa*) grown at room (RT) or high (HT) temperature. Values represent the mean ± standard deviation of three (mature plants) or six (microgreens, seedlings) biological replicates and four technical replicates. An asterisk (*) indicates a significant difference between plants grown at HT and RT within the same developmental stage or organ at the mature stage (Student’s *t*-test, *p* ≤ 0.05), while different letters indicate significant differences among developmental stages or organs grown under the same temperature conditions (one-way ANOVA, Duncan’s test, *p* ≤ 0.05).

In our study, we investigated the impact of HTs on the concentration of pigments and how the concentration changed during plant development and in different plant organs at the mature stage. In general, HTs had detrimental effects on the concentration of pigments (chlorophyll *a*, chlorophyll *b*, carotenoids, and porphyrins) in all analyzed tissues, with two exceptions (Figure 6). The first exception was that the slight decrease of 2% in the concentration of chlorophyll *a* in the leaves of mature broccoli grown in HTs was not statistically significant. The stability of the concentration of chlorophyll *a* in mature leaves could mean that mature leaves have some mechanisms that help them protect chlorophyll *a*, maybe through increased antioxidant activity. Nevertheless, the second exception was also observed in the leaves of mature broccoli grown in HTs. Interestingly, the concentration of carotenoids in the leaves of mature broccoli grown in HTs increased by 101% compared to the value in mature leaves grown at RT. Since carotenoids act as photoprotective pigments that can diminish the effect of ROS produced in HT conditions, their increase in the leaves of mature broccoli exposed to HTs could be a protective mechanism of mature leaves to protect chlorophyll *a*, the most important pigment for photosynthesis [65].

On the other hand, the observed decrease in the concentration of pigments in other tissues suggests detrimental effects of HTs on broccoli. The decline in pigment concentration with HT was previously reported for kale [28], cabbage [28,66], Indian mustard [67,68], Pakchoi [69], and watermelon [70]. The detrimental effects on chlorophyll *a* and *b* concentrations were more pronounced in mature broccoli heads than at younger stages (microgreens and seedlings). Given that the main function of broccoli heads is the production of seeds [71], it is possible that this organ is more prone to pigment degradation. The concentration of chlorophyll *b* was more diminished by HTs than chlorophyll *a* in all analyzed tissues, suggesting a higher susceptibility of chlorophyll *b* to HTs, which we observed in our earlier works [39,40]. A similar response was observed in kale and cabbage plants, in which the concentration of chlorophyll *b* was decreased under conditions of 32 °C compared to plants grown at 20 °C, while the concentration of chlorophyll *a* was not affected [28]. Chlorophyll *a*/*b* was increased by HTs in all analyzed stages and organs in stress conditions [72], although, in microgreens, the increase was not statistically significant. The most pronounced increase in chlorophyll *a*/*b* under HT conditions was in mature heads, indicating stress conditions and senescence [73].

In terms of the concentration of pigments at different developmental stages and organs at the mature stage, the lowest was observed in broccoli heads. Since the main function of broccoli heads is reproduction, it was expected that this organ would have a lower concentration of pigments involved in photosynthesis. On the other hand, the highest concentration of pigments was found at the microgreens stage and in the leaves of mature broccoli.

### 2.6. Oxidative Stress Parameters

**Figure 7:**
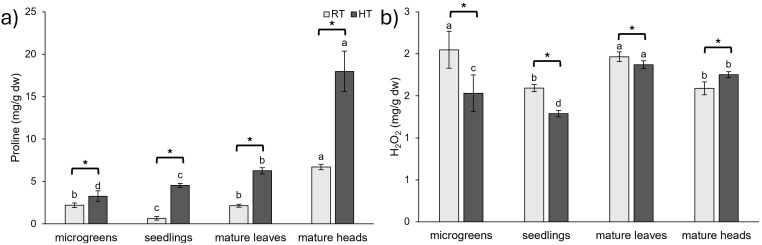
The concentration of (**a**) proline and (**b**) H_2_O_2_ during development and in different organs at the mature stage of broccoli (*Brassica oleracea* var. *cymosa*) grown at room (RT) and high (HT) temperature. Values represent the mean ± standard deviation of three (mature plants) or six (microgreens, seedlings) biological replicates and four technical replicates. An asterisk (*) indicates a significant difference between plants grown at HT and RT within the same developmental stage or organ at the mature stage (Student’s *t*-test, *p* ≤ 0.05), while different letters indicate significant differences among developmental stages or organs grown under the same temperature conditions (one-way ANOVA, Duncan’s test, *p* ≤ 0.05).

Proline acts as an osmoregulatory molecule and is often associated with various stressful conditions [74]. Our study revealed that broccoli plants cultivated under HT conditions exhibited a higher proline content across all developmental stages and organs compared to those grown at RT (Figure 7a). The most substantial increase of 587% was observed in broccoli seedlings. In the mature broccoli, proline levels increased by 191% in the leaves and 168% in the heads, while, in the microgreens, the increase was 47% compared to RT. Proline is commonly regarded as a biomarker of stress, and similar trends were reported for other species. For instance, blueberry seedlings exposed to HTs of 39 °C showed increased proline accumulation, and this effect was further enhanced by pretreatment with ferulic acid [53]. Similarly, elevated proline levels in Indian mustard plants (*B. junacea*) exposed to HTs (30 °C and 40 °C) were observed, and the increase was amplified by salicylic acid pretreatment [67]. Proline accumulation was also linked to heat tolerance in other Brassicaceae species. In a heat-tolerant line of Chinese cabbage (‘268’), exposure to temperatures of 40 °C for 8 days resulted in a progressive increase in proline levels, while there was no significant change observed in the heat-sensitive ‘334’ line [49]. These findings underscore the role of proline in conferring the resilience of plants to HT stress.

The highest proline concentration was observed in mature broccoli heads, particularly those grown at HT, suggesting that elevated proline levels may play a crucial role in maintaining osmotic balance and protecting this reproductive organ, both under normal and stress conditions.

In contrast, the concentration of hydrogen peroxide (H_2_O_2_) was generally lower in plants exposed to HT, except in the mature broccoli heads, where an increase in H_2_O_2_ concentration was observed (Figure 7b). The lower concentration of H_2_O_2_ in broccoli grown under HT conditions may have been a result of the activation of the plant’s antioxidant system, which reduced ROS production. Additionally, plants under HT conditions may have adjusted their metabolic processes, reducing H_2_O_2_ production to minimize oxidative stress and protect cellular components. The marked variations in how each developmental stage reacted to HTs most likely reflect underlying physiological and metabolic differences. Due to their early growth stages, microgreens and seedlings have higher cell division rates, less differentiated tissue, and increased metabolic flexibility, which may enable a more dynamic response to stress. Mature heads and leaves, on the other hand, have more specialized structures and metabolisms (such as complex tissue architecture and a high accumulation of storage compounds), which may limit their capacity to quickly adapt to heat stress. Divergent oxidative stress responses are also likely caused by variations in source–sink metabolism, energy demands, and antioxidant enzyme activities between stages. Future research should include lipid peroxidation markers (like malondialdehyde or 4-hydroxynonenal) and other ROS to provide a more complete oxidative stress profile.

### 2.7. Antioxidant Capacity

Vegetables are a great source of antioxidants, which help plants and animals maintain a desirable oxidant status [75]. Plant constituents that are highly involved in the neutralization of reactive oxygen species and radicals are phenolic compounds and vitamins [76]. In our study, we investigated the effect of HTs and plant development stage on antioxidant capacity by three methods (ABTS, DPPH, FRAP).

**Figure 8:**
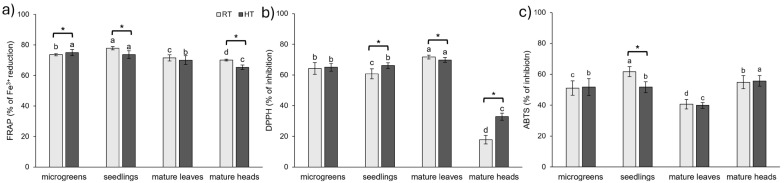
The antioxidant capacity measured by the (**a**) FRAP, (**b**) DPPH, and (**c**) ABTS methods during development and in different organs at the mature stage of broccoli (*Brassica oleracea* var. *cymosa*) grown at room (RT) or high (HT) temperature. Values represent the mean ± standard deviation of three (mature plants) or six (microgreens, seedlings) biological replicates and four technical replicates. An asterisk (*) indicates a significant difference between plants grown at HT and RT within the same developmental stage or organ at the mature stage (Student’s *t*-test, *p* ≤ 0.05), while different letters indicate significant differences among developmental stages or organs grown under the same temperature conditions (one-way ANOVA, Duncan’s test, *p* ≤ 0.05).

The results highlighted significant differences in antioxidant capacity across methods, developmental stages, and temperature treatments (Figure 8). The FRAP method revealed significant differences in antioxidant capacity in all analyzed tissues between the RT and HT groups, except in the leaves of mature broccoli. In most cases, HTs reduced Fe^3+^ reduction capacity, except in broccoli microgreens, where an increase was observed (Figure 8a). The DPPH method also showed differences between the RT and HT groups across most tissues, except those found in broccoli microgreens. In broccoli seedlings and mature heads, HTs increased the antioxidant capacity measured by the DPPH method, while leaves of mature broccoli grown under HT conditions exhibited lower antioxidant capacity than those grown at RT (Figure 8b). The ABTS method, however, was less effective in distinguishing between RT and HT, with the only statistically significant difference observed in broccoli seedlings, where HTs reduced antioxidant capacity (Figure 8c). In contrast to our findings, the temperature of 32 °C increased antioxidant capacity measured by the ABTS method in cabbage and kale [28] compared to plants grown at 20 °C. On the other hand, a heat shock of 45 °C and 50 °C (1 h/day for a week) had a negative impact on the antioxidant capacity of one-month-old tomato plants measured by the DPPH and ABTS methods [36]. In contrast, sprouts of wild radish grown at 30 °C had a higher antioxidant capacity measured by the DPPH method than those grown at 20 °C [14]. However, there was no statistical change in the antioxidant capacity of edible radish sprouts exposed to HTs [14]. Conversely, antioxidant capacity measured by the DPPH method decreased in *Gynura* leaves and stems exposed to 35 °C compared to 20 °C [77]. When *Gynura* plants were exposed to 25 °C, leaves exhibited increased antioxidant capacity, while stems exhibited decreased antioxidant capacity compared to plants grown at 20 °C [77]. In micropropagated *Lavandula viridis* and *Thymus lotocephalus*, antioxidant activity measured by the ABTS, DPPH, and FRAP methods increased with the rise in temperature, while the opposite trend was observed in *in vitro* cultures of these species [47].

In broccoli grown at RT, the seedlings exhibited the highest antioxidant capacity in both ABTS and FRAP assays. The lowest antioxidant activity measured by ABTS was found in the leaves of mature broccoli, while FRAP showed the lowest Fe^3+^ reduction capacity of broccoli heads. Interestingly, ABTS revealed a relatively high antioxidant capacity in broccoli heads under both RT and HT conditions, contrasting with the FRAP and DPPH results, where broccoli heads consistently showed the lowest antioxidant activity potential among the tissues analyzed. In the DPPH assay, mature leaves had the highest antioxidant capacity, but the same extract showed the lowest antioxidant activity using the ABTS method and the second lowest using FRAP (only slightly higher than in the broccoli heads). Together, these findings show that the antioxidant capacity markedly differs between developmental stages under RT conditions. For comparison, the antioxidant capacity of broccoli (*Brassica oleracea* var. *italica* cv. ‘Legacy’) sprouts, assessed using the DPPH method, was highest in 5-day-old and 11-day-old sprouts, while the lowest value was observed in 3-day-old sprouts [37]. Interestingly, 8-day-old sprouts exhibited intermediate antioxidant capacity, lower than both 5-day-old and 11-day-old sprouts but still higher than 3-day-old sprouts [37]. In contrast, in choy sum, the highest DPPH value was observed in 15-day-old plants (with three true leaves), followed by 7-day-old plants (with the first true leaf), with the lowest DPPH value found in 30-day-old plants (mature stage) [23].

In the HT group, younger developmental stages, i.e., microgreens and seedlings, did not differ from each other in their antioxidant capacity measured by the ABTS, DPPH, and FRAP methods. Similar to the RT group, the DPPH method recorded the greatest antioxidant potential for the leaves of mature broccoli and the lowest for the heads. In contrast, the ABTS method showed the highest value for the heads and the lowest for the leaves of mature broccoli. The FRAP method showed a decrease in antioxidant capacity in mature plants compared to younger developmental stages, with broccoli heads exhibiting the lowest values.

The ABTS method is effective in assessing both hydrophilic and lipophilic antioxidants, as well as high-pigment compounds, due to its ability to operate in both aqueous and organic solvents [78]. In contrast, the DPPH assay favors hydrophobic antioxidants, which are more likely to interact with the lipophilic DPPH radical [78]. The radically lower antioxidant capacity observed in broccoli heads using the DPPH method, compared to the other tissues, and the relatively high antioxidant capacity measured by ABTS could be attributable to the differing nature of antioxidants present in these tissues. Possibly, heads of broccoli contain a higher proportion of hydrophilic antioxidants that cannot be assessed adequately by the DPPH method. Indeed, heads of broccoli had the highest concentration of *L*-ascorbic acid, which is a hydrophilic antioxidant (Figure 3), and the lowest amount of lipophilic carotenoids (Figure 6c).

Altogether, these results suggest that the antioxidant profile of broccoli is shaped by developmental stage, organ, and temperature, with notable differences depending on the assay used. Under RT conditions, seedlings exhibited the highest antioxidant activity according to both FRAP and ABTS assays. In contrast, under HT conditions, FRAP indicated the highest activity of microgreens and seedlings, while ABTS showed the highest activity of mature heads. According to the DPPH assay, leaves of mature broccoli had the highest antioxidant capacity under both RT and HT conditions. These findings provide complementary insights into the antioxidant dynamics in broccoli across developmental stages, organs and environmental conditions.

### 2.8. Statistical Analysis

#### 2.8.1. Two-Way Factorial ANOVA

In order to assess the relative contribution of developmental stage or organ at mature stage and temperature to the variation in the analyzed parameters, as well as their potential interactive effects, we conducted a two-way factorial ANOVA. The obtained η^2^ values provided insights into the dominant factor shaping the metabolic responses of broccoli plants throughout development under different temperature conditions, and they are represented in Table 1.

The results of a two-way factorial ANOVA showed that developmental stage (and organ at the mature stage) had a significant impact on all 29 analyzed variables. Based on the measured parameters, the average contribution of developmental stage to the variance was 75%. Moreover, it explained more than 50% of the variance for 24 analyzed variables and over 90% of the variance for 12 analyzed variables. These 12 variables included antioxidant capacity measured by the DPPH method (97%), proline (96%), *L*-ascorbic (96%) and ferulic (95%) acid, kaempferol (95%), total carotenoids (94%), chlorophyll *a* (94%), total chlorophyll and carotenoids (93%), total chlorophyll (93%), porphyrins (93%), chlorophyll *b* (92%), and chlorophyll *a*/*b* ratio (91%).

Temperature, on the other hand, had a significant effect on 23 of the analyzed variables. On average, temperature explained 39% of the variance in these 23 variables. The highest proportion of variance caused by temperature was observed for proline (92%), followed by kaempferol (80%), chlorophyll *a*/*b* (76%), soluble sugars (73%), total flavonoids (65%), antioxidant capacity measured by DPPH (58%), and chlorophyll/carotenoid ratio (56%). Interestingly, temperature explained more variance than developmental stage for the concentration of soluble sugars, total hydroxycinnamic acids, and total tannins, which indicates an important role of these metabolite groups in the response of broccoli to HTs.

The interaction between developmental stage and temperature had a significant impact on 25 out of 29 analyzed variables. Based on the measured parameters, the average contribution of this interaction to the variance was 47% for these 25 variables. Interestingly, the interaction of developmental stage and temperature explained more variance than developmental stage alone for the concentration of total proanthocyanidins, hydroxycinnamic acids, and phenolic acids.

#### 2.8.2. Hierarchical Clustering

**Figure 9:**
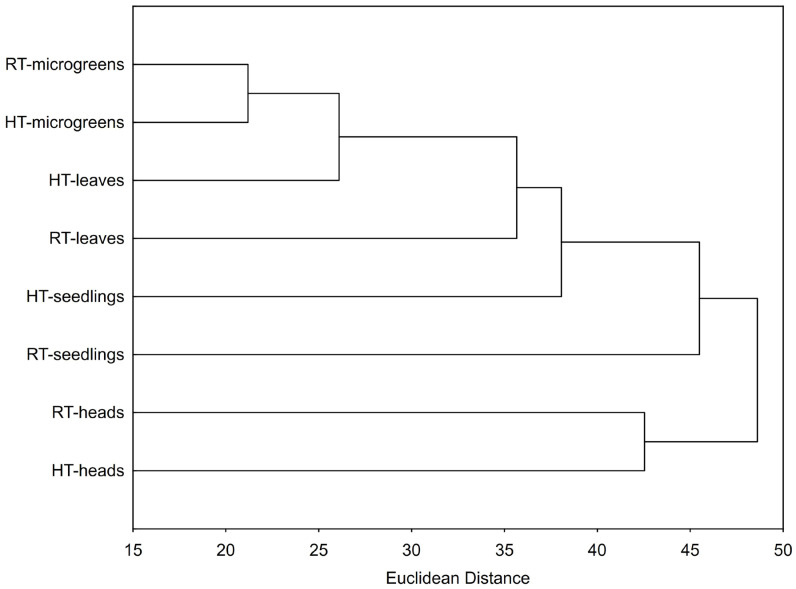
Hierarchical clustering of broccoli’s (*Brassica oleracea* var. *cymosa*) developmental stages and organs at the mature stage grown at room (RT) and high (HT) temperature based on measured groups and individual phytochemicals, pigments, oxidative stress parameters, and antioxidant capacity.

Hierarchical clustering analysis showed groupings of samples according to developmental stage (and the organ at the mature stage) and temperature conditions (Figure 9). The two temperature groups of the microgreens stage were the nearest, with the smallest differences caused by temperature. Within the developmental stage, the two temperature groups of seedlings were the most distant. This suggests that the seedlings most significantly reacted to HTs. Out of all developmental stages/organs, heads of mature broccoli were the most distant from the others, indicating biochemical differences of this organ in comparison to other developmental stages.

#### 2.8.3. Principal Component Analysis

**Figure 10:**
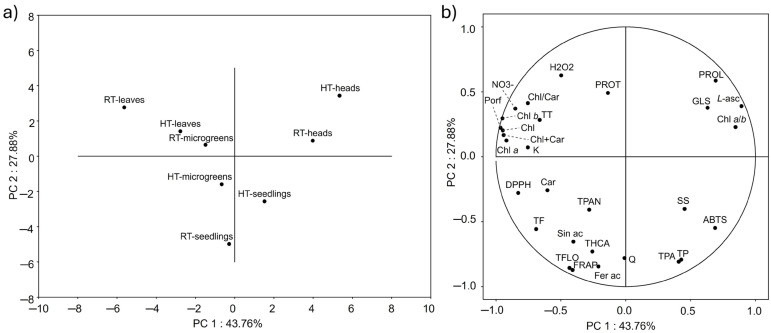
The principal component analysis showing (**a**) the relationship between different developmental stages and organs at the mature stage of broccoli (*Brassica oleracea* var. *cymosa*) grown at room (RT) and high (HT) temperature based on the analyzed variables, with the grouping displayed in part (**b**) of the figure. ABTS = 2,2′-azino-bis(3-ethylbenzothiazoline-6-sulfonic acid); Car = carotenoids; Chl = chlorophyll; DPPH = 2,2-diphenyl-1-picrylhydrazyl; Fer ac = ferulic acid; FRAP = ferric ion-reducing antioxidant power; GLS = total intact glucosinolates; H_2_O_2_ = hydrogen peroxide; HT = high temperature; K = kaempferol; *L*-asc = *L*-ascorbic acid; NO_3_^−^ = total nitrates; Porf = porphyrins; PROL = proline; PROT = soluble proteins; Q = quercetin; RT = room temperature; Sin ac = sinapic acid; SS = soluble sugars; TF = total flavonoids; TFLO = total flavonols; THCA = total hydroxycinnammic acids; TP = total phenolics; TPA = total phenolic acids; TPAN = total proanthocyanidins; TT = total tannins.

Principal component analysis was used to further understand the causal relationship between variables and samples (Figure 10). The first two principal components (PC 1 and PC 2) explained 72% of the total variance. Concentrations of pigments, *L*-ascorbic acid, kaempferol, nitrates, and antioxidant capacity measured by the DPPH method were the variables that contributed the most to PC 1. The heads and leaves of mature broccoli were on the opposite sides of PC 1 while being on the same side of PC 2, which indicated an opposite response in the variables that contributed the most to PC 1. Indeed, the leaves of mature broccoli had quite high concentrations of pigments and antioxidant capacity measured by the DPPH method, while heads had the lowest values of these variables (Figure 6 and Figure 8). The opposite could be seen in the concentration of *L*-ascorbic acid, which was the highest in the heads of mature broccoli and the lowest in the leaves of mature broccoli (Figure 3). On the other hand, antioxidant capacity measured by the FRAP method, the content of different groups of phenolic compounds (total flavonols, total phenolic acids, total phenolic compounds, and total hydroxycinnamic acids), and the concentration of ferulic acid and quercetin were the variables that contributed the most to PC 2. PC 2 primarily separated the microgreens and seedlings on one side and the mature broccoli samples (both heads and leaves) on the other side. The variables that contributed the most to PC 2 were antioxidant capacity measured by the FRAP method, the content of different groups of phenolic compounds (total flavonols, total phenolic acids, total phenolic compounds, and total hydroxycinnamic acids), and the concentration of ferulic acid and quercetin. Seedlings of both temperature groups, as well as microgreens grown in HTs, had higher concentrations of metabolites that contributed the most to PC 2 (Figure 1 and Figure 2).

The projection of the samples onto the PCA plot confirmed the findings of the hierarchical clustering analysis. Broccoli heads were clearly separated from other developmental stages, while leaves and microgreens were closer together, with moderate differences caused by temperature. This further confirmed that developmental stage plays a key role in defining the metabolic profile of broccoli, while temperature can modify specific parameters within the same stage.

#### 2.8.4. Pearson’s Correlation Coefficient

In the calculation of Pearson’s correlation coefficient, we used the mean values of each group to analyze the relationship between various groups and individual phytochemicals, pigments, oxidative stress parameters, and antioxidant capacity measured by three assays during the development of broccoli plants that were grown at RT or HT.

The results revealed that certain phytochemical compounds had strong positive and negative correlations, according to Meghanathan [79], which indicated complex interactions between the measured variables (Table S1). Total phenolic compounds and phenolic acids were very highly negatively correlated with the concentration of H_2_O_2_ (*r* = −0.82 and *r* = −0.82, respectively), which indicated the protective role of these metabolites in reducing oxidative stress-induced damage. Similarly, total flavonols and hydroxycinnamic acids, as well as sinapic acid, were very highly positively correlated with the antioxidant capacity measured by the FRAP assay, which further supports the role of phenolic compounds as antioxidants. Interestingly, proline was very highly negatively correlated with the antioxidant capacity measured by the FRAP assay, which could indicate that proline does not directly contribute to the antioxidant capacity of broccoli but rather plays a role in stress mitigation through other mechanisms, such as maintaining osmotic balance. Similarly, glucosinolates and *L*-ascorbic acid showed a very high negative correlation (*r* = −0.80 and *r* = −0.91, respectively) with the antioxidant capacity measured by the DPPH assay. This could reflect the nature of the DPPH radical, which prefers hydrophobic antioxidants over hydrophilic antioxidants, like *L*-ascorbic acid. In contrast, carotenoids, which are hydrophobic photoprotective pigments, exhibited a high positive correlation with the results of the DPPH assay, which further supports the preference of DPPH radicals for hydrophobic antioxidants. A high negative correlation between *L*-ascorbic acid and total flavonoids (*r* = −0.77), as well as a negative correlation between proline and total flavonoids, flavonols, and hydroxycinnamic acid (*r* = −0.80, *r* = −0.84, and *r* = −0.76, respectively), indicated that these compounds are tightly regulated in response to environmental and developmental changes. Furthermore, both chlorophyll a and b, as well as the total chlorophyll content, were highly negatively correlated with the concentration of *L*-ascorbic acid (*r* = −0.79, *r* = −0.73, and *r* = −0.77, respectively), while chlorophyll *a*/*b* was very highly positively (*r* = 0.88) correlated with the concentration of *L*-ascorbic acid. Higher concentrations of chlorophyll *a*/*b* are normally an indicator of stressful conditions [72], and these relationships may imply a protective role of *L*-ascorbic acid in mitigating stress effects.

These findings provide valuable insights into how broccoli’s phytochemical composition is influenced by both its developmental stage and environmental conditions, particularly temperature, which can modulate the balance between growth, defense, and antioxidant potential.

## 3. Materials and Methods

### 3.1. Plant Material

The seeds of broccoli, *Brassica oleracea* L. convar. *botrytis* (L.) Alef. var. *cymosa* Duch. (known as broccoli Calabrais), article number 424430, were obtained from the International Seeds Processing GmbH (Quedlinburg, Germany). Seeds were sown in sterile, soil-filled pots and placed in a climate chamber Fito-Clima 600 PLH (Aralab, Rio de Mouro, Portugal) under controlled conditions, with a 16/8 h photoperiod and 65% relative humidity, at room temperature (RT, 23 °C day/18 °C night). After five days, one group of plants continued to grow under the same conditions, while the other was exposed to high temperature treatment (HT, 38 °C day/33 °C night). Plants were regularly monitored and watered and collected at different developmental stages: microgreens (13 days after sowing) and seedlings (27 days after sowing). The experiment was conducted twice, once in the autumn of 2024 and once in the late winter of 2024. Plants were collected by cutting below the leaves and lyophilized.

A group of plants intended for the mature stage were grown in a field in raised beds until the mature stage was reached. When they reached a stage of mature head and leaves, plants were transferred into pots and put into the climate chamber Fito-Clima 600 PLH (where they were treated with HT (38 °C day/33 °C night) or RT (23 °C day/18 °C night) conditions) for the next five days. A longer period of treatment was avoided due to the start of senescence. After collecting leaves and heads, they were frozen in liquid nitrogen, lyophilized, and ground into a powder. Three biological replicates were weighed from each cultivation for each developmental stage/organ and temperature group.

### 3.2. Extraction of Phytochemicals

Broccoli extracts at a concentration of 30 mg/mL were prepared by mixing 70% ethanol (*v*/*v*) and lyophilized plant tissue powder. The mixture was rotated on a digital tube rotator (Thermo Scientific, Shanghai, China) at 30 rpm for 60 min at room temperature. After the incubation, the extracts were centrifuged at 18 000 rpm for 10 min using a MIKRO 220 centrifuge (Hettich, Westphalia, Germany). The obtained supernatants were stored at −20 °C until further analysis.

### 3.3. Measurement of Different Groups of Polyphenolic Compounds

UV/VIS spectrophotometry was used to measure different groups of phenolic compounds in extracts of different developmental stages and different organs at the mature stage exposed to RT and HT conditions, applying different colorimetric methods as follows. The measurements were conducted using a FLUOstar Optima microplate reader (BMG LABTECH, Ortenberg, Germany). For the blank, 70% ethanol was used instead of the extract.

For the determination of total phenolic compounds, a method with the Folin–Ciocalteu reagent was applied [80]. The amount of total phenolics in the samples was calculated indirectly based on the calibration curve of standard gallic acid (GA) solutions of known concentrations (0.08–2.50 mg/mL). The results were expressed as milligrams of GA equivalents per gram of dry weight (mg GAE/g dw).

The determination of total flavonoids followed the AlCl_3_ method [81]. The absorbance was measured at 520 nm. Total flavonoid content was calculated indirectly based on the calibration curve generated from standard quercetin (Q) solutions of known concentrations (0.04–2.50 mg/mL). The results were expressed as mg QE/g dw.

Total phenolic acids were estimated using the Arnow method described in Czigle et al. [82]. The absorbance was measured at 485 nm and the phenolic acid content was calculated indirectly based on the calibration curve of standard caffeic acid (CA) solutions of known concentrations (0.05–1.60 mg/mL). The results were expressed as mg CAE/g dw.

The measurement of total flavonols and hydroxycinnamic acids was conducted following the method described by Howard et al. [83], using diluted ethanolic extracts (6 mg/mL). Total flavonol content was calculated indirectly based on the calibration curve of standard Q solutions with known concentrations (0.02–0.31 mg/mL). Hydroxycinnamic acid content was calculated indirectly based on the calibration curve of standard CA solutions with known concentrations (0.05–1.60 mg/mL). The results were expressed as mg QE/g dw for total flavonols and CAE/g dw for total hydroxycinnamic acids.

To determine total tannin content, the method described by Sangeetha & Vedasree [84] was followed. The absorbance was measured at 740 nm and the total tannin content was calculated using a calibration curve prepared with standard GA solutions of known concentrations (0.01–1.30 mg/mL). The results were expressed as mg GAE/g dw.

The proanthocyanidins (condensed tannins) content was determined following the method described by Weidner et al. [85]. The absorbance was measured at 485. The total proanthocyanidins were calculated using a calibration curve prepared with standard catechin (Kat) solutions of known concentrations (0.02–1.25 mg/mL). The results were expressed as mg KatE/g dw.

### 3.4. Measurement of Individual Polyphenolic Compounds and L-Ascorbic Acid

Individual polyphenolic compounds and *L*-ascorbic acid were analyzed in hydrolyzed extracts. For that purpose, the extracts underwent hydrolysis with 1.2 M HCl for two hours at 80 °C and 300 rpm. An Agilent 1100 Series system equipped with a UV/VIS detector was used for analysis. Separation was performed on a Poroshell 120 SB-C18 non-polar column (4.6 × 75 mm, 2.7 µm particle size) with a Zorbax Extend-C18 guard column (4.6 × 12.5 mm, 5 µm particle size). The chromatographic conditions for separation, identification, and quantification followed those described by Šola et al. [17]. The mobile phase consisted of solvent A (0.2% acetic acid, *v*/*v*) and solvent B (0.2% acetic acid/80% methanol, *v*/*v*). The flow rate was set at 1 mL/min, and 50 µL of each sample was injected. Absorbance detection was performed at multiple wavelengths: 254 nm for *L*-ascorbic acid, 310 nm for ferulic and sinapic acids, and 360 nm for quercetin and kaempferol. Chromatograms were processed using ChemStation software B.04.03-SP1 (Agilent Technologies, Santa Clara, CA, USA). Compounds were identified based on retention times, with UV spectra compared to commercial standards. Quantification was performed using calibration curves, constructed from a mixed standard solution at known concentrations ranging from 0.05 to 0.25 mg/mL. The results were expressed as milligrams of individual component/g dw.

### 3.5. Measurement of Soluble Sugars

The measurement of total soluble sugars followed the method described by Dubois et al. [86], using diluted ethanolic extracts (1.20 mg/mL). The absorbance was measured at 485 nm. The concentration of total soluble sugars was calculated indirectly based on the calibration curve generated from standard sucrose (Suc) solutions of known concentrations (0.05–1.00 mg/mL). The results were expressed as mg SucE/g dw.

### 3.6. Measurement of Nitrogen-Containing Phytochemicals

Total soluble protein concentration was determined using the method described by Bradford [87]. The extracts in a concentration of 15 mg/mL were prepared in cold 100 mM phosphate buffer containing 0.1 mM EDTA (pH 7). The absorbance was measured at 595 nm. Phosphate buffer containing EDTA was used in place of the extract as a blank. Protein concentrations were calculated from a standard curve based on bovine serum albumin (BSA) solutions (0.06–2.00 mg/mL). The results were expressed as mg BSAE/g dw.

Total nitrate content was measured in ethanolic extracts according to Catalado et al. [88]. The absorbance was measured at 405 nm. The concentration of total nitrates was calculated using the calibration curve generated from standard KNO_3_ of known concentrations (0.03–2.00 mg/mL). The results were expressed as mg NO_3_^−^/g dw.

The content of total intact glucosinolates was determined in the extracts of a concentration of 15 mg/mL prepared in hot 70% methanol (90 °C), following the method described by Aghajanzadeh et al. [89]. The absorbance was measured at 405 nm. A blank was prepared using heated 70% methanol instead of the extract. The concentration of total intact glucosinolates in the samples was calculated indirectly based on the calibration curve generated from standard sinigrin (Sin) aqueous solutions of known concentrations (0.10–1.00 mg/mL). The results were expressed as mg SinE/g dw.

### 3.7. Measurement of Photosynthetic Pigments

Photosynthetic pigments were measured in an 80% acetone extract at a concentration of 10 mg/mL. The absorbance of the diluted extracts (5 mg/mL) was measured at 470, 575, 590, 628, 647, and 663 nm in a cuvette on a Nanodrop 2000c spectrophotometer (Thermo Fisher Scientific Inc., Waltham, MA, USA). The concentration of chlorophyll *a*, chlorophyll *b*, total carotenoids, and porphyrins was calculated using equations described in Sumanta et al. [90]. The results were expressed in mg/g dw.

### 3.8. Measurement of Oxidative Stress Parameters

The proline content was evaluated using a method from Ljubej et al. [91] in ethanolic extracts. The absorbance was measured at 520 nm. The proline content was calculated indirectly based on the calibration curve of standard *L*-proline solutions of known concentrations (0.02–0.60 mg/mL). The results were expressed in mg/g dw.

The hydrogen peroxide (H_2_O_2_) content was measured according to Junglee et al. [92]. The absorbance was measured at 405 nm and the content was calculated indirectly based on the calibration curve of H_2_O_2_ of known concentrations (2.00–60.00 µg/mL). The results were expressed in mg/g dw.

### 3.9. Measurement of Antioxidant Capacity

The determination of antioxidant capacity was measured using the ABTS, DPPH, and FRAP assays described by Poljuha et al. [93]. For the ABTS and DPPH assays, extracts at a concentration of 30 mg/mL in 70% ethanol were employed, while, for the FRAP assay, extracts were diluted to a concentration of 6 mg/mL in 70% ethanol. For the blank, 70% ethanol was used instead of the extract.

In the FRAP assay, the absorbance (*Abs*) of the mixture was measured at 595 nm. The reduction of the (Fe(III)-TPTZ) complex (%) was determined using the following formula:

$$\%\;reduction=\left(\frac{{Abs}_{\mathrm{extract}}-{Abs}_{\mathrm{blank}}}{{Abs}_{\mathrm{extract}}}\right)\times 100$$

In the DPPH assay, the absorbance was measured at 520 nm, while in the ABTS assay the absorbance was measured at 740 nm. For both assays, the percentage of inhibition of radicals was calculated using the following formula:

$$\%\;inhibition=\left(\frac{{Abs}_{\mathrm{blank}}-{Abs}_{\mathrm{extract}}}{{Abs}_{\mathrm{blank}}}\right)\times 100$$

### 3.10. Statistical Analysis

Statistical analysis was conducted using the Statistica 14.0 program (TIBCO Software Inc., Palo Alto, CA, USA). To evaluate differences between the RT and HT groups across developmental stages, Student’s *t*-test was employed. Differences among developmental stages within the same temperature group were analyzed using one-way analysis of variance (ANOVA), followed by Duncan’s new multiple range test for post hoc multiple comparisons. Statistical significance was set at *p* ≤ 0.05. To determine the relative contribution of developmental stage and temperature to the variation in the analyzed parameters, as well as their potential interactive effects, we conducted a two-way factorial ANOVA. To explore the similarity or dissimilarity of samples based on their phytochemical and antioxidant properties, multivariate analyses, including principal component analysis (PCA) and hierarchical clustering (HC), were conducted using Euclidean distance and single-linkage clustering. Additionally, Pearson’s linear correlation coefficients were calculated to assess relationships between measured variables. In PCA and HC analysis, as well as for the calculation of Pearson’s linear correlation coefficients, the mean values of each group of samples were used.

## 4. Conclusions

As the global temperature is rising, it is important to understand how an increase in temperature impacts the composition of the food we consume. Although broccoli heads are recognized as the most consumed part of broccoli in human nutrition, there is an increase in the consumption of younger stages of broccoli plants. In this study, we have analyzed the impact of high growing temperatures on the phytochemical composition and antioxidant capacity of broccoli plants at different developmental stages as well as on two different organs at the mature stage. Analyses showed that developmental stage and organ at the mature stage play a dominant role in shaping the metabolic profile of broccoli, although temperature can modulate specific metabolic responses within each stage. The highest number of analyzed variables—26 out of the 29 analyzed—significantly impacted by HT was found for the mature broccoli heads. Among them, 19 variables were decreased and 7 were increased by HTs, with the most substantial changes being an increase in proline by 168%, an increase in chl *a*/*b* by 102%, and an inhibition of DPPH radicals by 84%. On the other hand, the lowest number of variables susceptible to HTs (66%) was found for the leaves of mature broccoli. Out of 19 parameters that were significantly affected by HT, 9 increased, while 10 decreased. The most notable change was an increase in soluble sugar concentration by 246%. Proline concentration increased by 191% and total carotenoids by 101%. In seedlings, HT significantly impacted 83% of the analyzed parameters, with 7 parameters being increased and 17 decreased. The most dramatic change observed in this study was an increase in proline concentration by 587% in seedlings. Additionally, soluble sugars increased by 128%. HT also had a significant impact on microgreens, affecting 69% of the analyzed parameters. Interestingly, microgreens had more increased (11) than decreased (9) variables in response to HT. The most substantial change was an increase by 66% in the concentration of ferulic acid, followed by a 47% rise in proline and a 47% rise in soluble sugars. These findings underscore the complexity of metabolic regulation in broccoli and emphasize the importance of considering both developmental stage and environmental conditions when assessing its nutritional and functional properties. Understanding these interactions could aid in optimizing growth conditions and selecting resilient cultivars for improved stress tolerance and nutritional quality. Determining the sensitivity of different developmental stages to HTs can aid in maximizing nutritional value during climate stress by guiding harvesting schedules and cultivation techniques. Furthermore, the observed rises in substances like proline and sugars in particular stages could be used as biochemical markers in breeding programs to choose genotypes that can withstand HTs. From a commercial standpoint, emphasizing how HTs cause microgreens or seedlings to produce more bioactive compounds could help promote them as functional foods with improved health benefits, potentially increasing their commercial appeal.

## Figures and Tables

**Table 1:** The contribution of developmental stage, temperature, and their interaction to variance (η^2^) based on two-way factorial ANOVA.

	Developmental Stage	Temperature	Stage × Temperature
TP	0.31 ***	0.01	0.13 *
TPA	0.12 ***	0.00	0.20 ***
THCA	0.21 ***	0.30 ***	0.49 ***
TF	0.82 ***	0.64 ***	0.70 ***
TFLO	0.81 ***	0.28 ***	0.52 ***
TT	0.07 *	0.17 ***	0.00
TPAN	0.47 ***	0.01	0.57 ***
K	0.95 ***	0.80 ***	0.76 ***
Q	0.79 ***	0.20 *	0.39 **
Sin ac	0.80 ***	0.07	0.10
Fer ac	0.95 ***	0.05	0.62 ***
*L*-asc	0.96 ***	0.43 **	0.41 **
SS	0.64 ***	0.73 ***	0.56 ***
PROT	0.80 ***	0.02	0.50 ***
NO_3_^−^	0.88 ***	0.13 *	0.67 ***
GLS	0.75 ***	0.08 *	0.16 *
Chl *a*	0.94 ***	0.20 ***	0.08 *
Chl *b*	0.92 ***	0.39 ***	0.24 ***
Car	0.94 ***	0.09 *	0.68 ***
Porf	0.93 ***	0.39 ***	0.20 ***
Chl	0.93 ***	0.30 ***	0.05
Chl + Car	0.93 ***	0.26 ***	0.04
Chl *a*/*b*	0.91 ***	0.76 ***	0.80 ***
Chl/Car	0.79 ***	0.56 ***	0.60 ***
PROL	0.96 ***	0.92 ***	0.86 ***
H_2_O_2_	0.67 ***	0.33 ***	0.47 ***
ABTS	0.66 ***	0.07 *	0.29 ***
DPPH	0.97 ***	0.58 ***	0.45 ***
FRAP	0.74 ***	0.27 ***	0.35 ***

Asterisk indicates significance levels: * *p* < 0.05; ** *p* < 0.01; *** *p* < 0.001. ABTS = 2,2′-azino-bis(3-ethylbenzothiazoline-6-sulfonic acid); Car = carotenoids; Chl = chlorophyll; DPPH = 2,2-diphenyl-1-picrylhydrazyl; Fer ac = ferulic acid; FRAP = ferric ion-reducing antioxidant power; GLS = total intact glucosinolates; H_2_O_2_ = hydrogen peroxide; K = kaempferol; *L*-asc = *L*-ascorbic acid; NO_3_^−^ = total nitrates; Porf = porphyrins; PROL = proline; PROT = soluble proteins; Q = quercetin; Sin ac = sinapic acid; SS = soluble sugars; TF = total flavonoids; TFLO = total flavonols; THCA = total hydroxycinnammic acids; TP = total phenolics; TPA = total phenolic acids; TPAN = total proanthocyanidins; TT = total tannins.

## Data Availability

Data are contained within the article and Supplementary Materials.

## Supplementary Materials

The following supporting information can be downloaded at https://www.mdpi.com/article/10.3390/plants14121825/s1: Figure S1: The concentration of total identified phenolics (TIP) during development and in different organs at the mature stage of broccoli (*Brassica oleracea* var. *cymosa*) grown at room (RT) or high (HT) temperature. Values represent the mean ± standard deviation of three (mature plants) or six (microgreens, seedlings) biological replicates. An asterisk (*) indicates a significant difference between plants grown at HT and RT within the same developmental stage or organ at the mature stage (Student’s *t*-test, *p* ≤ 0.05), while different letters indicate significant differences among developmental stages or organs grown under the same temperature conditions (one-way ANOVA, Duncan’s test, *p* ≤ 0.05). Figure S2: Comparison of direction and intensity of change (%) of variables among young and mature broccoli (*Brassica oleracea* var. *cymosa*) tissue types under the effect of high temperature. Car = carotenoids; Chl = chlorophyll; Fer ac = ferulic acid; GLS = total intact glucosinolates; K = kaempferol; *L*-asc = *L*-ascorbic acid; Porf = porphyrins; PROL = proline; PROT = soluble proteins; Q = quercetin; Sin ac = sinapic acid; SS = soluble sugars; TF = total flavonoids; TFLO = total flavonols; THCA = total hydroxycinnamic acids; TP = total phenolics; TPA = total phenolic acids; TPAN = total proanthocyanidins; TT = total tannins.; Table S1: Pearson’s correlation coefficients (*r*) between individual and groups of phytochemicals, soluble sugars, photosynthetic pigments, oxidative stress parameters, and antioxidant capacity of broccoli microgreens, seedlings, mature broccoli leaves, and heads. Car = carotenoids; Chl = chlorophyll; Fer ac = ferulic acid; GLS = total intact glucosinolates; K = kaempferol; *L*-asc = *L*-ascorbic acid; Porf = porphyrins; PROL = proline; PROT = soluble proteins; Q = quercetin; Sin ac = sinapic acid; SS = soluble sugars; TF = total flavonoids; TFLO = total flavonols; THCA = total hydroxycinnamic acids; TP = total phenolics; TPA = total phenolic acids; TPAN = total proanthocyanidins; TT = total tannins.

## Abbreviations

The following abbreviations are used in this manuscript:

ABTS	2,2′-azino-bis(3-ethylbenzothiazoline-6-sulfonic acid)
ANOVA	analysis of variance
BSAE	bovine serum albumin equivalent
CAE	caffeic acid equivalent
Car	carotenoid
Chl	chlorophyll
DPPH	2,2-diphenyl-1-picrylhydrazyl
dw	dry weight
Fer ac	ferulic acid
FRAP	ferric ion-reducing antioxidant power
GAE	gallic acid equivalent
GLS	total intact glucosinolates
HC	hierarchical clustering
HT	high temperature
K	kaempferol
KatE	catechin equivalent
*L*-asc	*L*-ascorbic acid
PCA	principal component analysis
Porf	porphyrins
PROL	proline
PROT	soluble proteins
Q	quercetin
QE	quercetin equivalent
ROS	reactive oxygen species
RT	room temperature
Sin ac	sinapic acid
SINE	sinigrin equivalent
SS	soluble sugars
SucE	sucrose equivalent
TF	total flavonoids
TFLO	total flavonols
THCA	total hydroxycinnamic acids
TP	total phenolics
TPA	total phenolic acids
TPAN	total proanthocyanidins
TT	total tannins
