# Supplementary figures and images for: Developmental and Temperature-Driven Variations in Metabolic Profile and Antioxidant Capacity of Broccoli (Brassica oleracea var. cymosa)
Source: Plants (Basel). 2025 Jun 13;14(12):1825. doi: 10.3390/plants14121825 (PMC12196942; doi:10.3390/plants14121825)

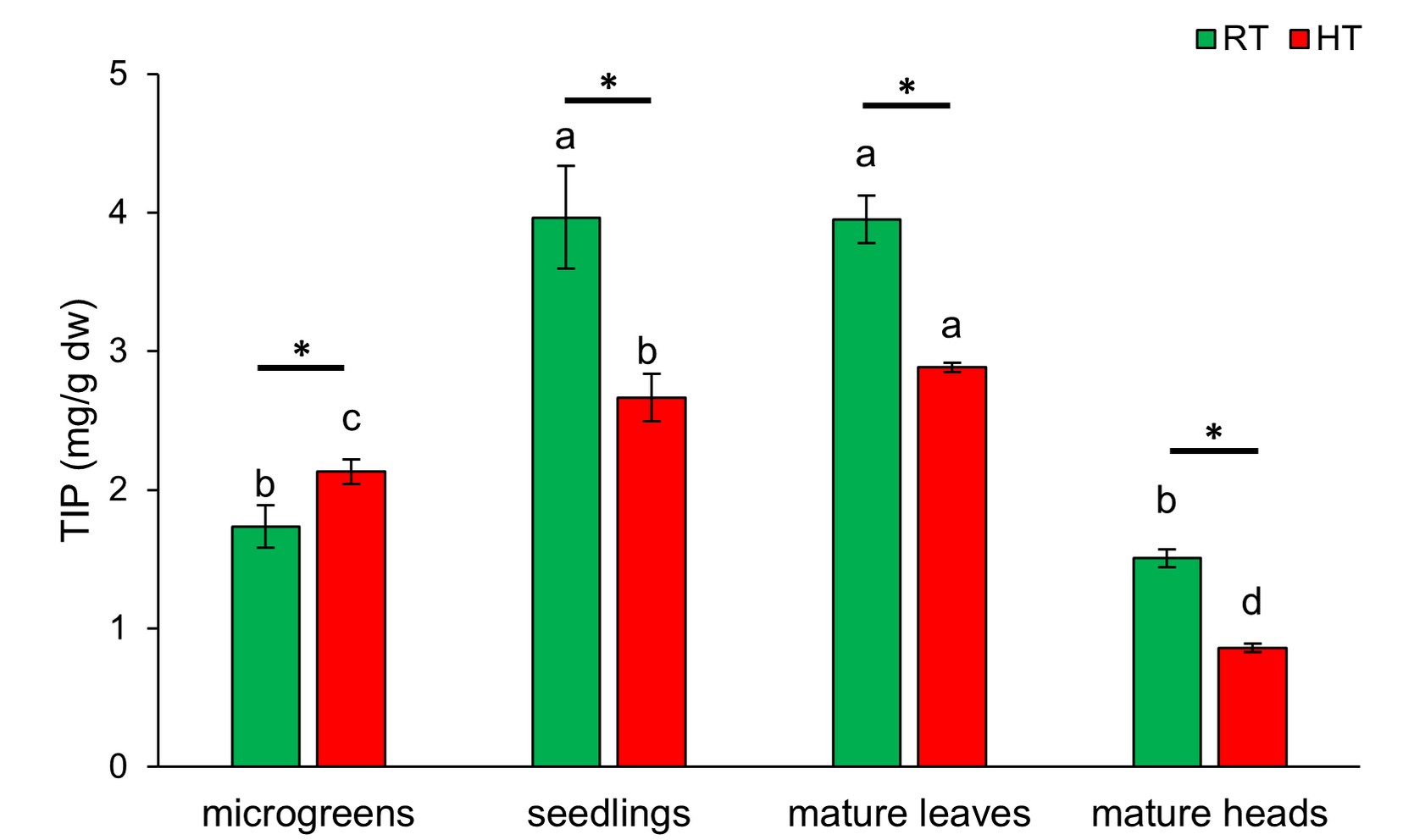

Supplement: Supplementary file 1 [file plants-14-01825-s001.zip › Figure S1.jpg]

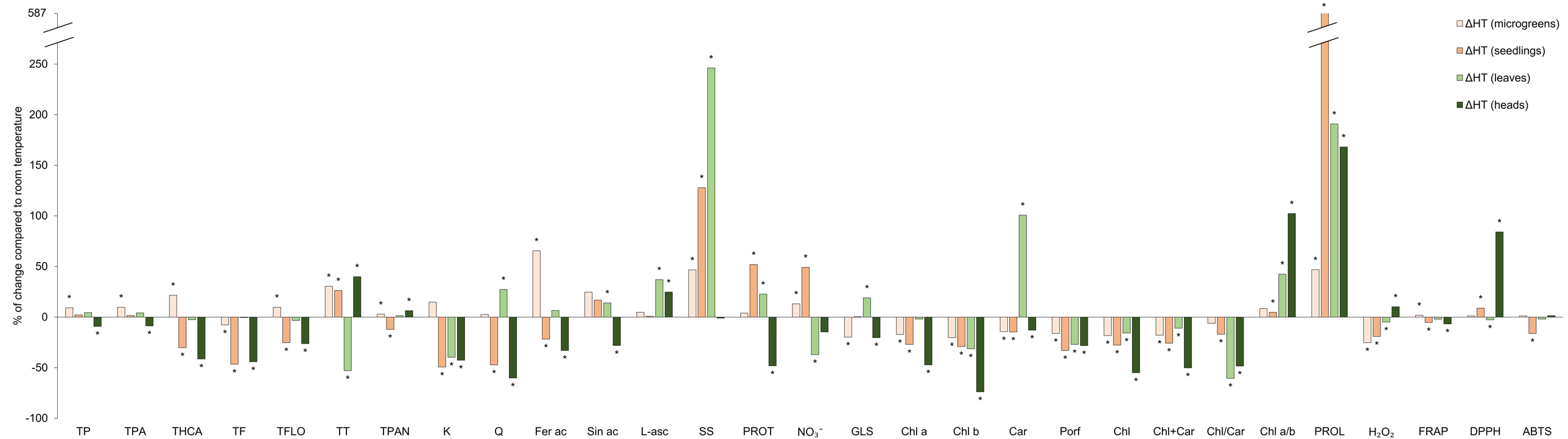

Supplement: Supplementary file 1 [file plants-14-01825-s001.zip › Figure S2.jpg]
